# A Central Role for Foxp3+ Regulatory T Cells in K-Ras-Driven Lung Tumorigenesis

**DOI:** 10.1371/journal.pone.0005061

**Published:** 2009-03-30

**Authors:** Courtney A. Granville, Regan M. Memmott, Andria Balogh, Jacopo Mariotti, Shigeru Kawabata, Wei Han, Jaclyn LoPiccolo, Jason Foley, David J. Liewehr, Seth M. Steinberg, Daniel H. Fowler, M. Christine Hollander, Phillip A. Dennis

**Affiliations:** 1 Medical Oncology Branch, Center for Cancer Research, National Cancer Institute, Bethesda, Maryland, United States of America; 2 Experimental Transplantation and Immunology Branch, Center for Cancer Research, National Cancer Institute, Bethesda, Maryland, United States of America; 3 Biostatistics and Data Management Section, Center for Cancer Research, National Cancer Institute, Bethesda, Maryland, United States of America; New York University School of Medicine, United States of America

## Abstract

**Background:**

K-Ras mutations are characteristic of human lung adenocarcinomas and occur almost exclusively in smokers. In preclinical models, K-Ras mutations are necessary for tobacco carcinogen-driven lung tumorigenesis and are sufficient to cause lung adenocarcinomas in transgenic mice. Because these mutations confer resistance to commonly used cytotoxic chemotherapies and targeted agents, effective therapies that target K-Ras are needed. Inhibitors of mTOR such as rapamycin can prevent K-Ras-driven lung tumorigenesis and alter the proportion of cytotoxic and Foxp3+ regulatory T cells, suggesting that lung-associated T cells might be important for tumorigenesis.

**Methods:**

Lung tumorigenesis was studied in three murine models that depend on mutant K-Ras; a tobacco carcinogen-driven model, a syngeneic inoculation model, and a transgenic model. Splenic and lung-associated T cells were studied using flow cytometry and immunohistochemistry. Foxp3+ cells were depleted using rapamycin, an antibody, or genetic ablation.

**Results:**

Exposure of A/J mice to a tobacco carcinogen tripled lung-associated Foxp3+ cells prior to tumor development. At clinically relevant concentrations, rapamycin prevented this induction and reduced lung tumors by 90%. In A/J mice inoculated with lung adenocarcinoma cells resistant to rapamycin, antibody-mediated depletion of Foxp3+ cells reduced lung tumorigenesis by 80%. Likewise, mutant K-Ras transgenic mice lacking Foxp3+ cells developed 75% fewer lung tumors than littermates with Foxp3+ cells.

**Conclusions:**

Foxp3+ regulatory T cells are required for K-Ras-mediated lung tumorigenesis in mice. These studies support clinical testing of rapamycin or other agents that target Treg in K-Ras driven human lung cancer.

## Introduction

Lung cancer has been the leading cause of cancer deaths in American men since 1954 and in American women since 1987 [1], which reflects historical differences in smoking habits. Lung cancer remains a daunting problem that is mostly related to smoking, with over 215,000 new cases and 160,000 deaths expected in the US in 2008 [1]. Smoking is associated with resistance to cytotoxic chemotherapies and targeted therapies in lung cancer patients, and over 90 million current or former smokers in the United States are at permanent increased risk to develop lung cancer [2]. Thus, there is great need to understand and mitigate the effects of smoking as it relates to the development and treatment of lung cancer.

Activating mutations in K-Ras have been identified in approximately 25% of human lung adenocarcinomas that are primarily associated with smoking [3]–[5]. In preclinical models, K-Ras mutations are observed in over 90% of lung tumors induced by the tobacco-specific carcinogen 4-methylnitrosamino-1-(3-pyridyl)-1-butanone (NNK). Oncogenic K-Ras stimulates activation of the Akt/mTOR pathway, which contributes to the development of lung tumors [6], [7]. The FDA-approved immunosuppressant, rapamycin, as well as its analogues, are mTOR inhibitors, and this class of drugs decreases K-Ras induced lung tumorigenesis in mice. We recently reported that rapamycin, when administered to achieve physiologically relevant trough levels, reduced NNK-induced lung tumorigenesis in A/J mice by 90% [8]. These results are consistent with studies of transgenic models of prostate, breast and lung cancer, where treatment with rapamycin or rapamycin analogues prevented or reversed premalignant lesions [7], [9], [10]. Thus, early steps of tumorigenesis in many mouse models of cancer are reliant on mTOR activation. Despite the promise of mTOR inhibition as a preventive approach, little is known about the mechanisms underlying its efficacy.

Activation of K-Ras in the mouse lung generates an inflammatory process. In A/J mice, inflammation is highly associated with susceptibility to NNK-induced lung tumorigenesis. In mice genetically engineered to express mutant K-Ras, mTOR inhibition has been shown to reduce inflammatory processes in the lung [7]. The immunosuppressive properties of mTOR inhibitors, coupled with their efficacy for tumor prevention in mouse models, suggested that modulation of the immune system is important for mutant K-Ras mediated lung tumorigenesis. Regulatory T cells (Treg) suppress autoreactive T cells, and thus prevent autoimmunity [11]. Treg are a subset of CD4+ T cells and express the transcription factor, Foxp3 [12]. Preclinical studies suggest that Treg may play an important role in limiting the development of an effective immune response against cancer [13]. Tumor-associated Treg have been observed in lymphomas and human cancers of the lung, ovary, breast, prostate, and colon [14], [15]. In lung cancer, Treg are increased in tumor tissue relative to surrounding normal lung tissue and are associated with an increased risk of recurrence [15]. Treg also regulate the ability of inhibitors of cyclooxygenase-II to decrease lung tumorigenesis in xenograft and viral models of lung tumorigenesis [16]. Because rapamycin can alter T cell function and prevent the development of NNK-induced lung tumors that are associated with K-Ras mutations, we hypothesized that modulation of immune function might be an important determinant of lung tumorigenesis. Our studies show that rapamycin unexpectedly counteracts an increase in lung-associated Treg by NNK that precedes the detection of tumors. Depletion of Treg using an antibody or genetic deletion also greatly diminished lung tumorigenesis in other K-Ras driven mouse models, suggesting a new strategy that might have utility for lung cancers characterized by mutations in K-Ras.

## Methods

### Mice

A/J and Foxp3^sf/+^ mice were obtained from Jackson Laboratories (Bar Harbor, ME) at 5 weeks of age and housed according to the guidelines of the Animal Care and Use Committee of the National Institutes of Health (NIH). K-Ras^LA2^ mice were obtained from the Mouse Models of Human Cancer Consortium and housed according to the same guidelines. For the NNK study, 10 6-week old female A/J mice per group were given three 100 mg/kg doses of NNK or saline (EaglePicher Phamaceuticals, Lenexa, KS) as previously described [8]. IO33 cells were a generous gift of Dr. Steven Belinsky (U. of New Mexico Cancer Center) and 1×10^5^ cells were injected via tail vein in 100 µl of normal saline into 6-week old A/J females. Genetically modified mice were genotyped by polymerase chain reaction as described previously according to published protocols (http://mouse.ncifcrf.gov for Kras^LA2^ and JAX.org for Scurfy).

Rapamycin was obtained from LC Laboratories (Woburn, MA) and injected at 1.5 mg/kg every other day following a 4.5 mg/kg loading dose as previously described. For IO33 studies, mice were treated for 2 weeks with rapamycin following the same dosing schedule, or anti-CD25 antibody (Harlan Biosciences) or rat non-immune IgG1 (Sigma) (0.5 mg 2×/wk for week 1, followed by 0.5 mg/wk) that was begun one week prior to IO33 injection.

### Enumeration of Splenic Cells

Cells were analyzed by three-color flow cytometry on a FACSCalibur (BD Biosciences, San Jose, CA) instrument using CellQuest software (BD Biosciences). Intracellular flow cytometry using the APC anti-mouse/rat Foxp3 staining set from eBioscience (San Diego, CA) was used according to manufacturers instructions. Five to ten thousand live events were acquired for analysis, with propidium iodide exclusion of dead cells.

### Immunohistochemical and immunoblotting analyses

Detection of Foxp3 and CD3 cells by IHC was performed using the FJK-16s anti-mouse/rat Foxp3 antibody from eBioscience (San Diego, CA) and the anti-human CD3 antibody from DAKO (Carpinteria, CA) using the manufacturer's instructions. IHC analysis of lung tissues from mice treated with rat IgG or anti-CD25 antibodies was performed using a biotinylated anti-mouse/rat Foxp3 antibody (eBioscience, clone FJK-16s). Numbers of lung infiltrating cells were assessed on a single lung section from 5 mice/group. Immunoblotting for Foxp3 expression in human and murine lung cell lines was performed using the eBio7979 anti-mouse/human Foxp3 antibody from eBiosciences (San Diego, CA) using the manufacturer's recommendation.

### Statistical Analyses

Comparisons between two groups were performed using a Wilcoxon rank sum test. A Jonckheere-Terpstra trend test was used to determine the association of Foxp3 expressing cells with the number of tumors. All p-values are two-tailed and have not been adjusted for multiple comparisons.

## Results

### Tobacco carcinogen exposure increases lung associated Foxp3+ cells

**Figure 1:**
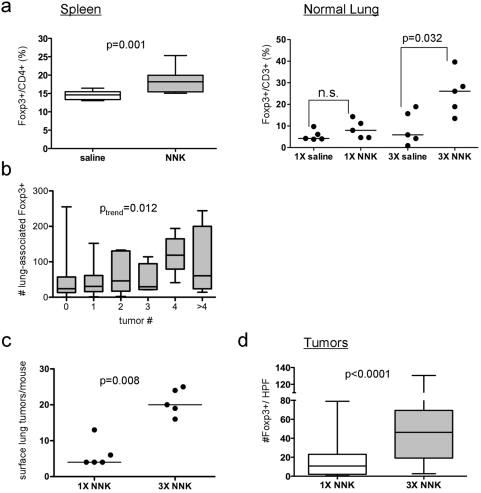
NNK increases Foxp3+ cells. (a) Assessment of Foxp3+ cells in splenic and lung tissues. One week after NNK exposure, Foxp3+ T cell subsets were assessed in spleens (left) as a fraction of total CD4+ splenocytes and in lungs (right) as a fraction of total CD3+ cells. (b) Correlation of number of NNK-induced lung tumors with number of Foxp3+ cells in surrounding lung tissues. Tumors were counted in mice 16 wk after NNK administration, and scoring for Foxp3+ cells was performed using IHC. A trend for the number of Foxp3+ cells in surrounding normal lung with the number of tumors per lung is shown. (c–d) Dose-dependent induction of lung tumors (c) and Foxp3+ cells (d) by NNK. For (a, b, and d), boxes indicate interquartile range, lines indicate median, and whiskers indicate minimal and maximal values. For (a and c), each point represents a mouse and the line represents the median. A high-powered field (HPF) indicates a field under 100× magnification.

A/J mice were treated with NNK, and immune cell number and function were characterized in splenocytes and lung tissues. One week after completion of three weekly doses of NNK, NNK decreased splenocyte number but not function (data not shown), and caused a 21% relative increase in splenic CD4+CD25+Foxp3+ regulatory T cells (Fig. 1a, left panel). Because regulatory T cells are associated with tumor tolerance, we hypothesized that a similar increase in Foxp3+ regulatory T cells in lung tissues may be associated with tobacco carcinogen-induced tumorigenesis. Immunohistochemistry was used to assess Foxp3+ cells and CD3+ cells in lung tissues from NNK-treated mice. One week after administration of either a single dose or three doses of NNK (and prior to the development of tumors), the fraction of lung associated Foxp3+/CD3+ cells increased 2- and 4-fold, respectively (Fig. 1a, right panel). Lung-associated Foxp3+ cells correlated with NNK-induced lung tumorigenesis, because 16 weeks after administration of NNK, there was a trend between the number of tumors per lung and the number of Foxp3+ cells in surrounding lung tissues (Fig. 1b). Induction of lung tumors and tumor-associated Foxp3+ cells by NNK was also dose-dependent. A single dose of NNK induced twice as many tumors and Foxp3+ cells as spontaneous tumors (data not shown). Mice that received three doses of NNK had nearly three times as many tumors and infiltrating Foxp3+ cells as mice that received one dose of NNK (Fig. 1c-d). Because expression of Foxp3 has been reported in lung epithelial cells and some tumor cell lines [17]–[19], we co-stained lung tissues or lymph nodes for Foxp3 and CD3 expression, and confirmed that expression of Foxp3 was observed in CD3+ cells but not epithelial cells (Fig. S1a–e). In addition, Foxp3 protein was not expressed in human lung cancer cell lines or cell lines derived from murine lung tumors (Fig. S1f).

### Agents that decrease Foxp3+ cells in lung tissues decrease lung tumorigenesis

**Figure 2:**
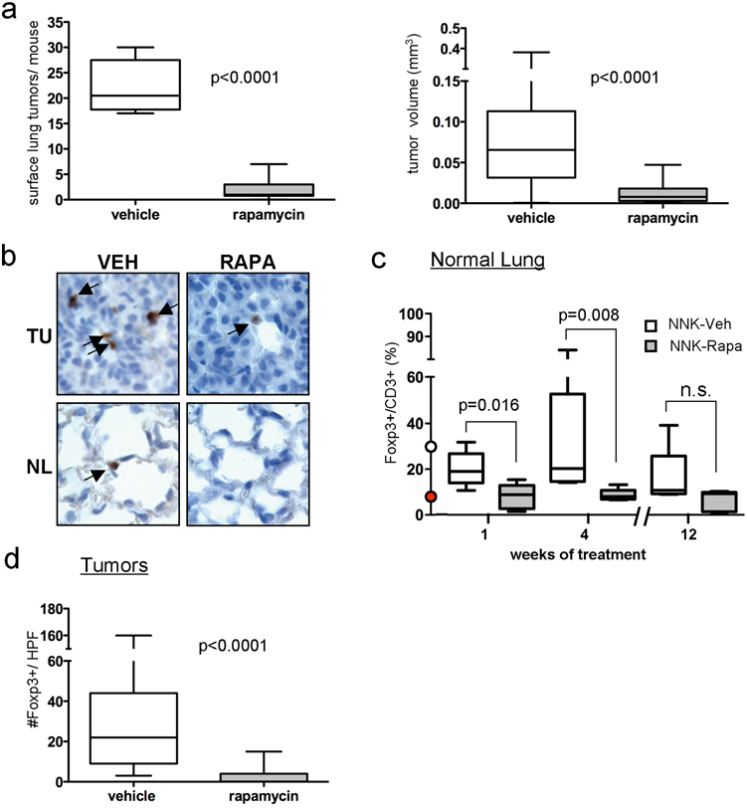
Rapamycin prevents NNK-induced tumorigenesis and depletes lung-associated Foxp3+ cells in NNK-treated mice. (a) Tumor multiplicity (left) and tumor size (right) after 12 wk of rapamycin or vehicle. (b) Representative staining for Foxp3+ cells in tumors (TU) and normal airway epithelium (NL). (c) The fraction of Foxp3+/total CD3+ cells in normal lung tissues from saline or NNK-exposed mice treated with rapamycin or vehicle for 1, 4 or 12 weeks was calculated after IHC was performed. The red and white dots at week 0 indicate the percentages of Foxp3+/CD3+ cells in the lungs of A/J mice prior to and after NNK administration, respectively. (d) The number of Foxp3+ cells in tumors arising 16 weeks after NNK exposure in the presence or absence of rapamycin was determined using IHC. For (a, c, and d), boxes indicate interquartile range, lines indicate median, and whiskers indicate minimal and maximal values. A high-powered field (HPF) indicates a field under 100× magnification.

Rapamycin inhibits lung tumorigenesis more effectively when it is administered prior to the detection of tumors. To assess whether this observation is related to lung-associated Foxp3+ cells, A/J mice were treated with NNK, and lungs and spleens were harvested at various time points after administration of rapamycin or vehicle. Consistent with earlier studies, rapamycin markedly decreased the size and multiplicity of NNK-induced lung tumors (Fig. 2a). mTOR was inhibited in tumor cells and normal airway epithelium (Fig. S2a), and steady state rapamycin levels were constant throughout the study. Rapamycin rapidly reversed the induction of lung-associated Foxp3+ cells by NNK, and maintained depletion of Foxp3+ cells throughout the study (Fig. 2c–d), even though splenic CD4+CD25+Foxp3+ cells were increased at 12 wk by rapamycin (Fig. S2b), consistent with previous results [20]. The depletion of lung-associated Foxp3+ cells preceded tumor development because the number of Foxp3-expressing cells was decreased by 84% after 1 week of treatment (when tumors were not visible). Although rapamycin also reduced the overall number of CD3+ T cells (data not shown), the Foxp3+/CD3+ ratio was depleted to levels observed in non-NNK exposed mice (Fig. 2c). Tumors that did arise in the presence of rapamycin had fewer infiltrating Foxp3+ cells than controls, even after accounting for their smaller size (Fig. 2d). Thus, NNK and rapamycin reciprocally regulate Foxp3+ cells in lung tissues prior to tumor development, suggesting that lung-associated Foxp3+ cells contribute to tumorigenesis.

**Figure 3:**
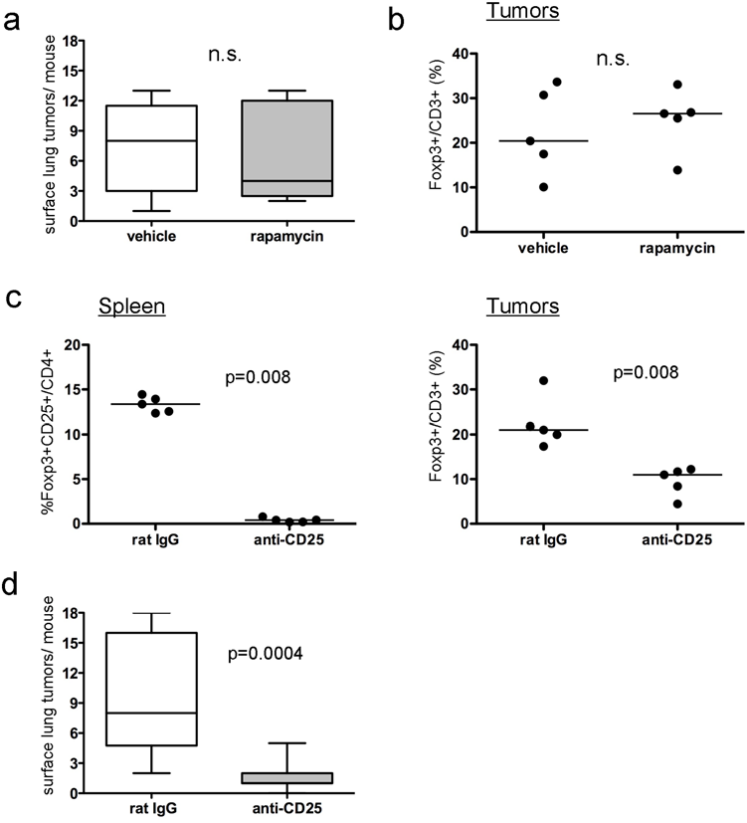
Depletion of Foxp3+ cells using an anti-CD25 antibody decreases the ability of rapamycin-resistant lung adenocarcinoma cells to form tumors. (a–b) Effect of rapamycin on IO33 tumor multiplicity (a) or tumor-associated percent of Foxp3+/CD3+ cells (b). (c) Effect of rat IgG or anti-CD25 antibodies on the number of splenic Foxp3+CD25+/CD4+ cells (left) and IO33 tumor-associated %Foxp3+/CD3+ cells (right). (d) Effect of anti-CD25 antibody or rat IgG on IO33 lung tumor multiplicity. For (a) and (d), boxes indicate interquartile range, lines indicate median, and whiskers indicate minimal and maximal values. For (b–c), each point represents a mouse and the line represents the median. n.s., not significant.

To test this hypothesis, an antibody against CD25 that can deplete Treg was administered to A/J mice after exposure to NNK, and lung tumorigenesis was assessed after 16 wk (data not shown). Although splenic CD25+ cells were rapidly decreased by 90% and maintained for the length of the study, lung associated Foxp3+ cells were only decreased transiently. After 16 wk, there was no inhibition of lung tumorigenesis and no decrease in lung associated Foxp3+ cells. Because long-term depletion of lung-associated Foxp3+ cells by anti-CD25 antibodies was not achievable, we developed a short-term assay, and performed a series of studies using syngeneic lung adenocarcinoma cell lines derived from NNK-induced tumors in A/J mice [20]. Relative to the other lung adenocarcinoma cell lines tested, IO33 cells were most resistant to inhibition of proliferation by rapamycin *in vitro* (Fig. S3a) despite inhibition of mTOR (Fig. S3b). When injected via tail vein, IO33 cells rapidly formed highly invasive lung adenocarcinomas in A/J mice (Fig. S3c). Treatment of IO33-injected mice with rapamycin had no effect on tumorigenesis (Fig. 3a) and did not affect the number of tumor-associated Foxp3+ cells (Fig. 3b), despite inhibition of mTOR in normal bronchial epithelium and IO33 tumors (Fig. S3d). Varying the sequence of rapamycin administration and cell injection did not increase responsiveness of the IO33 cells (data not shown). Because the anti-CD25 antibody decreased lung-associated Foxp3+ cells with short term-treatment, inoculation studies with IO33 cells were repeated, and A/J mice were treated with the anti-CD25 antibody prior to injection of IO33 cells. Treatment with anti-CD25 antibody decreased splenic Foxp3+CD25+ cells by 95% (Fig. 3c, left panel), decreased lung-associated Foxp3+ cells by 65% (Fig. 3c, right panel) and decreased the multiplicity of IO33 adenocarcinomas by 80% (Fig. 3d). These results are similar to the results observed with rapamycin in the studies using NNK.

### Genetic ablation of Foxp3+ cells decreases K-Ras mediated tumorigenesis

**Figure 4:**
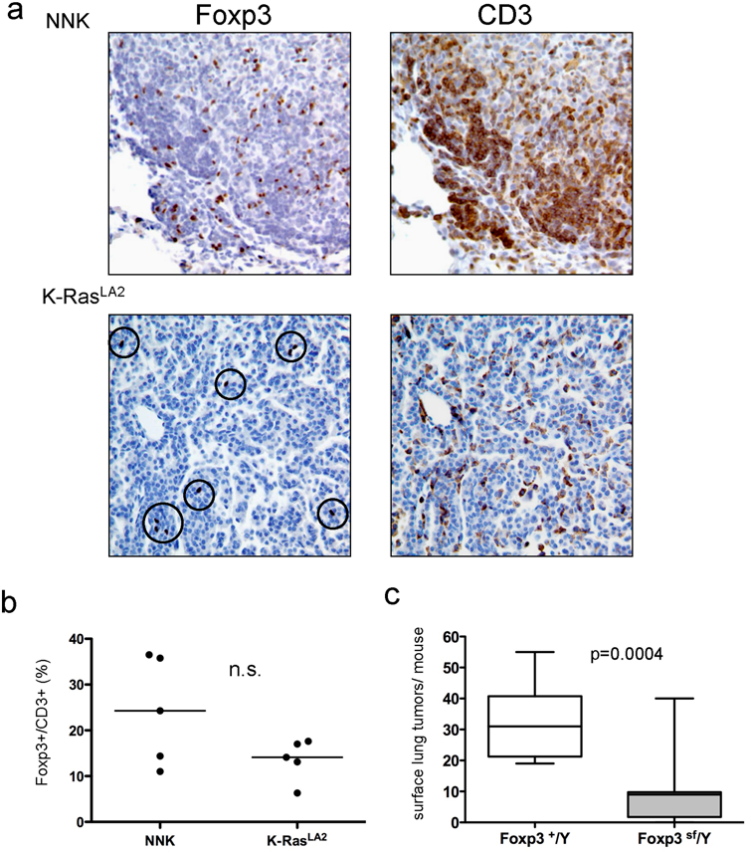
Genetically engineered mice that lack Foxp3+ cells develop fewer K-Ras driven lung tumors. (a) Representative IHC of tumor-associated Foxp3+ and CD3+ cells in tobacco-carcinogen (NNK) and K-ras^LA2^ transgenic mouse models of lung tumorigenesis. (b) Quantification of IHC analysis in (a) for tumor-associated percent of Foxp3+/CD3+ cells. Each point represents a mouse and the line represents the median; n.s., not significant. (c) Tumor multiplicity in K-RAS^LA2/wt^/Foxp3^−^/Y or K-RAS^LA2/wt^/Foxp3^+^/Y offspring. Boxes indicate interquartile range, lines indicate median, and whiskers indicate minimal and maximal values.

A comparison of tumor-associated Treg in the KRas^LA2^ model of lung cancer was performed to assess whether the induction of Foxp3+ cells was a general feature of K-Ras driven lung tumorigenesis or specific for NNK-induced tumors. K-Ras^LA2^ mice develop early-onset lung adenocarcinomas [22]. Foxp3+ and CD3+ cells were detected in each tumor type, and the overall number of Foxp3+ and CD3+ cells was lower in K-Ras^LA2^ mice than in mice treated with NNK (Fig. 4a). However, the fraction of Foxp3+/CD3+ cells was not statistically different between NNK-induced tumors and tumors from KRas^LA2^ mice (Fig. 4b). To confirm the role of Foxp3+ cells in this system, K-Ras^LA2^ mice were crossed to scurfy mice. Scurfy mice bear a loss-of-function mutation in the Foxp3 transcription factor (*Foxp3* ^sf/+^). Hemizygous males lack Foxp3+ cells and develop lethal autoimmunity that phenocopies the human IPEX syndrome [23], [24]. Because *Foxp3* is x-linked, lung tumorigenesis was assessed in 3-week-old *K*-Ras^LA2^Foxp3^sf^/Y males. Scurfy males were distinguishable from their wildtype littermates by their small size and scaly skin, but were not moribund at the time of sacrifice. Lymphocytic infiltrates were observed in lungs from scurfy males, although the severity varied widely (data not shown). Compared to *K-Ras^LA2^Foxp3* ^+^/Y males, K-*Ras^LA2^Foxp3* ^sf^/Y males developed 75% fewer tumors (Fig. 4c). These data confirm in a third model system that depletion of Foxp3+ cells inhibits K-Ras driven lung tumorigenesis.

## Discussion

Using carcinogen, allograft and transgenic models of lung cancer driven by K-Ras, we have shown that Foxp3+ cells are essential for lung tumorigenesis. The tissue specific induction of Foxp3+ cells in lung tissues prior to tumor development suggests that NNK-induced Foxp3+ T cells provide a permissive environment for the development of K-Ras-driven lung tumors. Such an environment may also be important for the development of lung metastasis, because smoking can double the metastatic burden in lungs in preclinical models [25].

A common feature linking smoking induced K-Ras mutations in human lung cancer and preclinical models driven by tobacco carcinogens that cause K-Ras mutations is inflammation. In both cases, the presence of Foxp3+ cells is likely important for limiting the extent of inflammation and tissue damage, albeit at a potential cost of promoting tumorigenesis. Smoking causes chronic obstructive pulmonary disease (COPD), which is an independent risk factor for lung cancer [26]. Bronchoalveolar lavage (BAL) fluid from patients with COPD or smokers contains increased Treg compared to BAL fluid from healthy never smokers [27]. Similarly, smoking is also associated with squamous cell carcinoma (SCC) of the oral cavity, and increased Foxp3+ cells have been observed in SCC compared to non-cancerous epithelial tissues [28]. NNK, the most prevalent tobacco carcinogen, alters many components of inflammation, including cytokine expression and cellular immunity, and the susceptibility of different mouse strains to NNK-induced lung tumorigenesis has been linked to differences in lung immunity [22]. One important alteration caused by NNK is induction of cyclooxygenase II (COX-2) activity. Inhibitors of COX-2 can decrease NNK-induced lung tumorigenesis [29], and Sharma *et al.* have shown that the ability of COX-2 inhibitors to decrease lung tumorigenesis (in models not related to tobacco) is dependent upon decreasing Foxp3+ cells [16].

Inflammation is also associated with lung tumorigenesis in transgenic mice that express mutant K-Ras in lung tissues. Our studies showed that the ratio of Foxp3+/CD3+ cells was not statistically different between the NNK model and the K-Ras^LA2^ model, but the total numbers of each cell type was increased in the tobacco carcinogen model. Eliminating Foxp3+ cells by crossing K-Ras^LA2^ mice to scurfy mice nonetheless decreased lung tumorigenesis, which shows that even though fewer tumor infiltrating lymphocytes were induced in the K-Ras^LA2^ model, Foxp3+ cells still played a critical role. The increased inflammatory response in lungs from scurfy mice that occurred as a result of loss of Foxp3+ cells may have decreased tumor growth through elimination of nascent tumors. Wislez *et al.* previously showed an important role for alveolar macrophages in mediating inhibition of tumor growth by high doses of a rapamycin analogue in a transgenic model of lung cancer similar to K-Ras^LA2^ (K-Ras^LA1^). However, alterations in Foxp3+ cells were not reported in this study. Because activated macrophages and tumor cells can recruit Foxp3+ cells [30], [31], it is possible that the role attributed to alveolar macrophages was mediated through activation of Foxp3+ cells. Together, these studies show that Foxp3+ cells provide a critical link between inflammation and cancer in multiple models of lung tumorigenesis driven by mutant K-Ras, and highlight the possibility that careful titration of lung- associated Foxp3+ cells might allow effective anti-tumor immunity without tissue damage from unabated inflammation.

Although these studies provide rationale to target Foxp3+ cells for the prevention or treatment of K-Ras-driven lung tumors, the responsiveness of Foxp3+ cells may depend on the agent utilized to target Foxp3+ cells and the transformation status of the cells that bear K-Ras mutations. For example, rapamycin selectively reduced the number of Foxp3+ cells induced by NNK in lung tissues prior to tumor development, thereby providing a reciprocal relationship with NNK, but had no effect when fully transformed IO33 adenocarcinoma cells were injected. Similar results were observed when rapamycin was administered after NNK-induced lung tumors were allowed to develop in A/J mice for 26 wk [8]. When administered from week 26 to week 32, rapamycin decreased tumor size by 50% but did not affect tumor multiplicity, and the number of tumor infiltrating Foxp3+ cells was not decreased (data not shown). The clinical experience with analogues of rapamycin that have been tested as cancer therapeutics is consistent with these preclinical observations, in that mTOR inhibitors only have modest activity as single agents in patients with advanced lung cancer [32], [33]. Taken together, these data suggest that rapamycin, which is an FDA-approved oral agent with a high therapeutic index, might be most effective as a preventative strategy primarily for smokers with premalignant lesions or carcinomas in situ that bear occult K-Ras mutations.

Aggressive and invasive K-Ras-induced adenocarcinomas (IO33 and K-Ras^LA2^) remained sensitive to more direct targeting of Foxp3+ cells through a neutralizing anti-CD25 antibody or genetic deletion. This indicates that direct Treg cell depletion strategies that are being evaluated clinically [34] could have therapeutic value in more advanced stages of K-Ras driven lung cancer. However, the fact that long-term administration of an anti-CD25 antibody was effective in splenocytes but not in lung tissues suggests that there may be tissue specificity for the ability of antibodies to deplete Foxp3+ cells and/or that compensatory mechanisms such as increases in CD25-/Foxp3+ cells occur with long-term use. Therefore, other therapeutic approaches may need to be developed to selectively deplete Foxp3+ cells in advanced K-Ras driven lung cancers. The development of multiple therapies that deplete Foxp3+ cells at different stages of K-Ras induced tumorigenesis might eventually provide new options for lung cancer treatment and prevention.

## Supporting Information

Figure S1 Foxp3 is expressed in CD3+ lymphocytes, but is not detectable in murine lung epithelium or lung epithelium-derived cultured cell lines. (a–e) Representative immunohistochemical co-staining of Foxp3 and CD3 in cells from NNK-induced A/J mice lung adenomas (a–c) and lung-associated lymph nodes (d–e). Foxp3 is blue/gray and CD3 is brown. (f) Immunoblotting analysis for Foxp3 expression in human and murine lung cell lines. Cells were treated with 100 nM rapamycin or vehicle for 24 hr to confirm lack of expression of Foxp3 in these cell lines and lack of inhibition of Foxp3 expression by rapamycin. H157 and A549 are human lung adenocarcinoma cell lines; HBEC are human bronchial epithelial cell lines immortalized with CDK4 and h-TERT with or without K-Ras mutations (KTR and KTC, respectively); hPBMCs are human peripheral blood mononuclear cells that were used as a positive control; IO33, CL13, CL25, and CL30 are lung adenocarcinoma cell lines derived from NNK-induced tumors in A/J mice.
(2.43 MB TIF)

Figure S2 Rapamycin inhibits mTOR in lung tissues and increases the fraction of Foxp3+/CD4+ splenocytes. (a) Representative IHC of phospho-S6 in normal lung (NL) and lung tumors (TU) 16 hours after the last dose of rapamycin in A/J mice. (b) During the tumorigenesis study, the effects of rapamycin versus vehicle on percent of splenocytes that were Foxp3+/CD4+ cells was assessed using FACS after 1, 4, and 12 weeks of treatment. The red and white dots at week 0 indicate the basal percent of splenic Foxp3+/CD4+ cells prior to and after NNK administration, respectively. Boxes indicate interquartile range, lines indicate median, and whiskers indicate minimal and maximal values.
(1.21 MB TIF)

Figure S3 IO33 cells are resistant to growth inhibition by rapamycin and form invasive lung tumors in A/J mice. (a) Dose-dependent inhibition of proliferation of murine and human lung cancer cell lines by rapamycin. In vitro, rapamycin only modestly inhibits proliferation of IO33 cells relative to other A/J-derived lung adenocarcinoma cell lines (CL30, CL25, and CL13) and human lung cancer cells (H1155). (b) Rapamycin inhibits mTOR in IO33 cells in vitro. mTOR inhibition was evaluated by immunoblotting analysis of cells treated with rapamycin for 2 h using antibodies specific for mTOR substrates, phospho-S6 and total 4E-BP1. (c) Syngeneic IO33 cells form invasive lung tumors in A/J mice when injected via tail vein. A representative whole mount of A/J lungs and heart 2 wk after tail vein injection with 106 IO33 cells is shown. Note multi-focal lung tumors and invasion into the ventricular wall. (d) Rapamycin inhibits mTOR in vivo, as assessed by IHC analysis of phospho-S6 in normal lung (NL) and IO33 lung tumors (TU) in A/J mice.
(3.21 MB TIF)
