# Supplementary figures and images for: A Central Role for Foxp3+ Regulatory T Cells in K-Ras-Driven Lung Tumorigenesis
Source: PLoS One. 2009 Mar 30;4(3):e5061. doi: 10.1371/journal.pone.0005061 (PMC2659439; doi:10.1371/journal.pone.0005061)

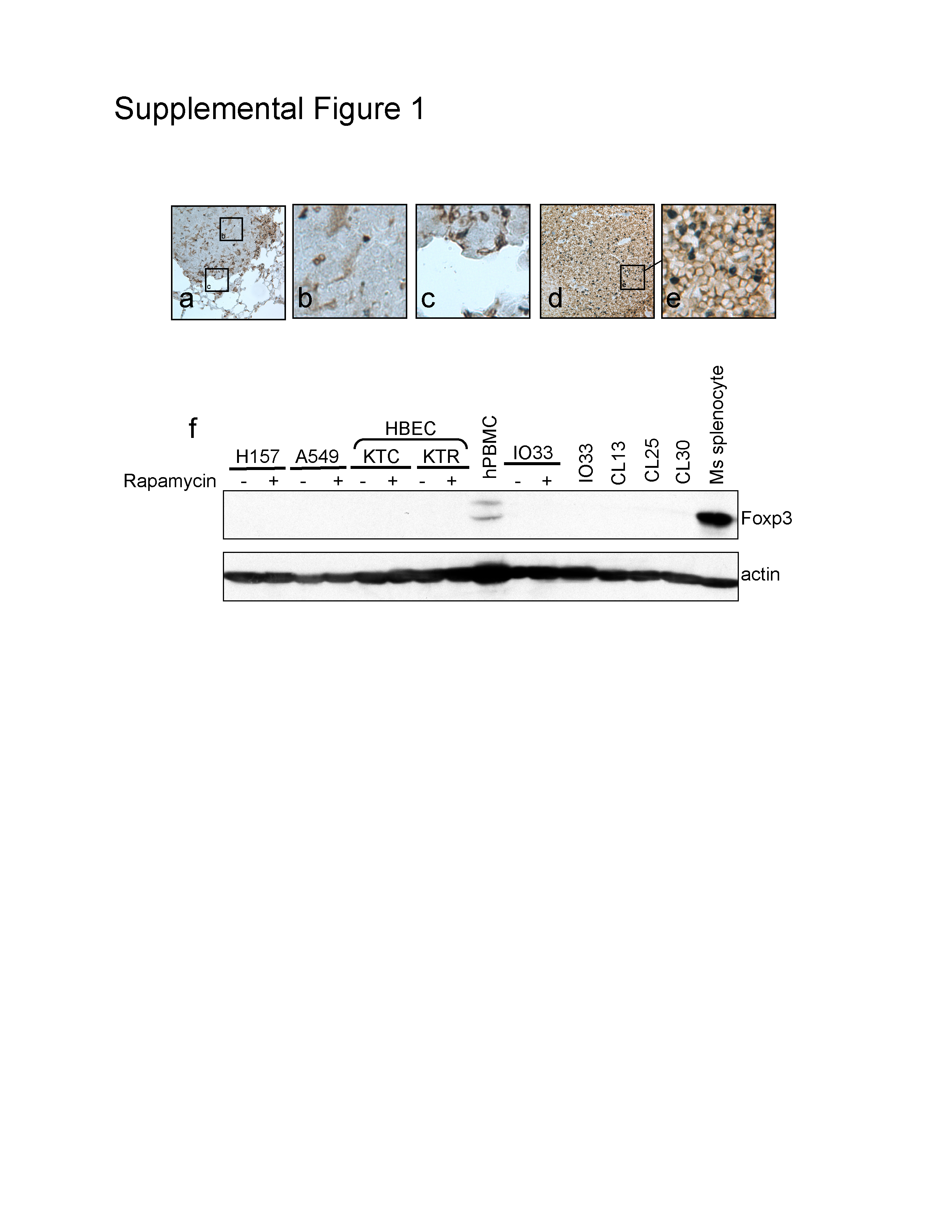

Supplement: Figure S1 — Foxp3 is expressed in CD3+ lymphocytes, but is not detectable in murine lung epithelium or lung epithelium-derived cultured cell lines. (a–e) Representative immunohistochemical co-staining of Foxp3 and CD3 in cells from NNK-induced A/J mice lung adenomas (a–c) and lung-associated lymph nodes (d–e). Foxp3 is blue/gray and CD3 is brown. (f) Immunoblotting analysis for Foxp3 expression in human and murine lung cell lines. Cells were treated with 100 nM rapamycin or vehicle for 24 hr to confirm lack of expression of Foxp3 in these cell lines and lack of inhibition of Foxp3 expression by rapamycin. H157 and A549 are human lung adenocarcinoma cell lines; HBEC are human bronchial epithelial cell lines immortalized with CDK4 and h-TERT with or without K-Ras mutations (KTR and KTC, respectively); hPBMCs are human peripheral blood mononuclear cells that were used as a positive control; IO33, CL13, CL25, and CL30 are lung adenocarcinoma cell lines derived from NNK-induced tumors in A/J mice. (2.43 MB TIF) [file pone.0005061.s001.tif]

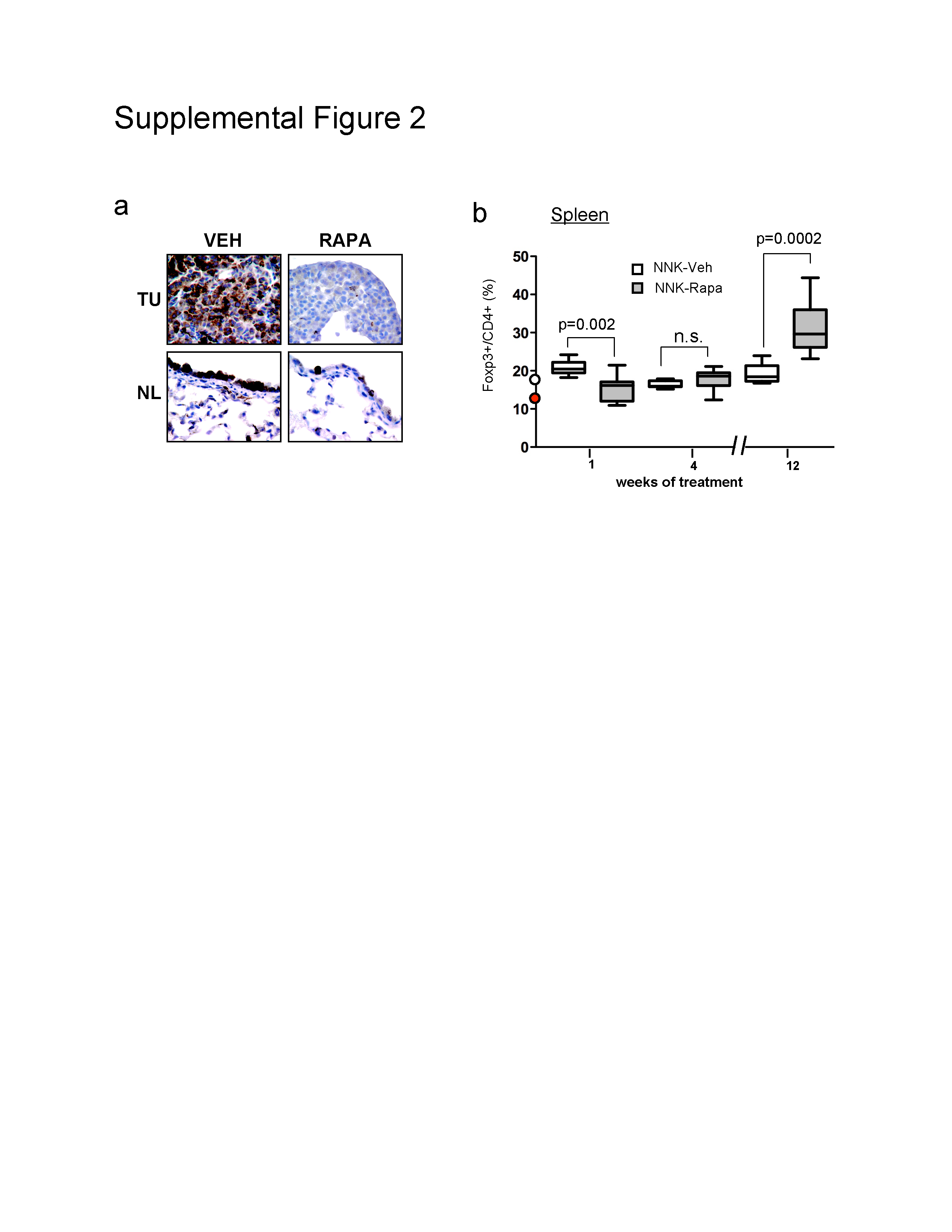

Supplement: Figure S2 — Rapamycin inhibits mTOR in lung tissues and increases the fraction of Foxp3+/CD4+ splenocytes. (a) Representative IHC of phospho-S6 in normal lung (NL) and lung tumors (TU) 16 hours after the last dose of rapamycin in A/J mice. (b) During the tumorigenesis study, the effects of rapamycin versus vehicle on percent of splenocytes that were Foxp3+/CD4+ cells was assessed using FACS after 1, 4, and 12 weeks of treatment. The red and white dots at week 0 indicate the basal percent of splenic Foxp3+/CD4+ cells prior to and after NNK administration, respectively. Boxes indicate interquartile range, lines indicate median, and whiskers indicate minimal and maximal values. (1.21 MB TIF) [file pone.0005061.s002.tif]

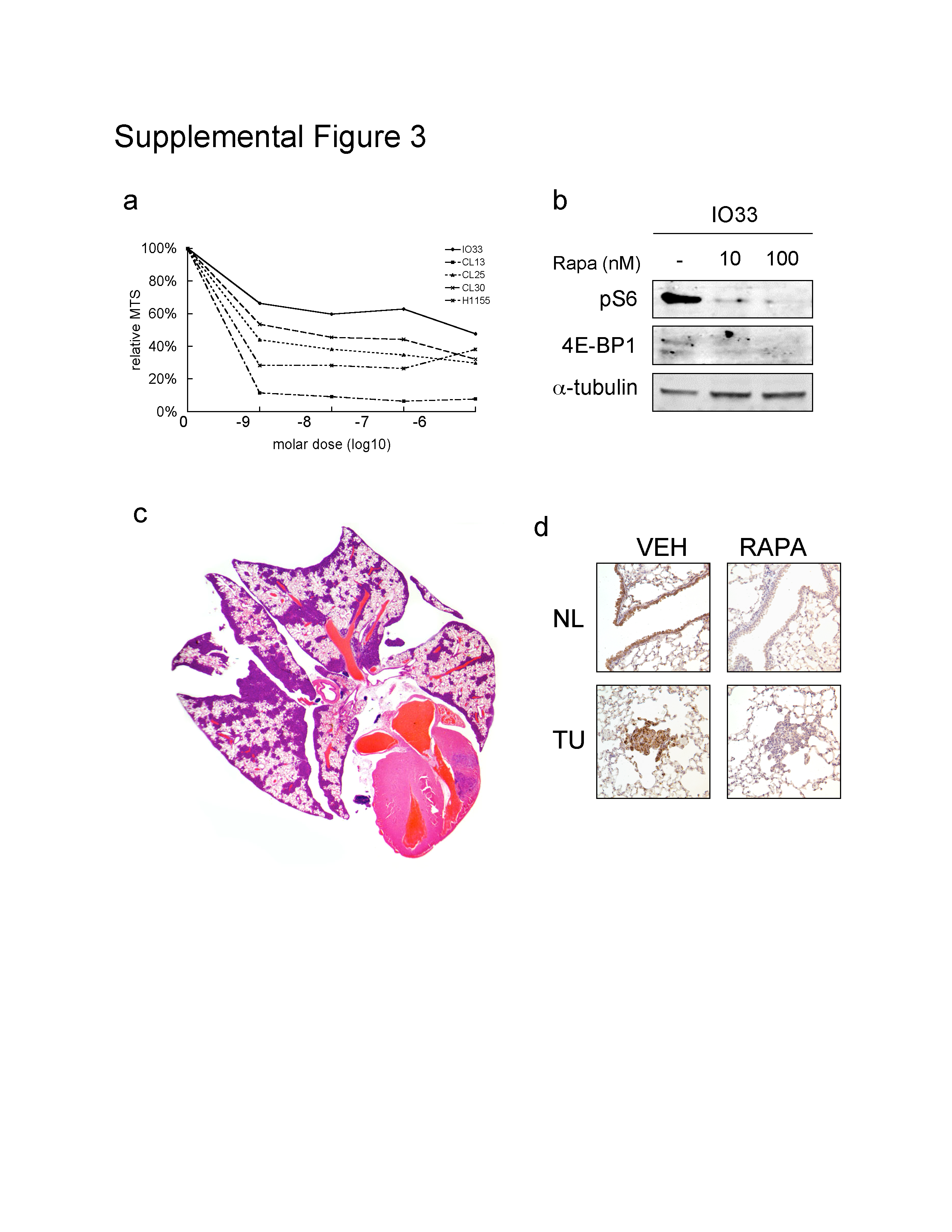

Supplement: Figure S3 — IO33 cells are resistant to growth inhibition by rapamycin and form invasive lung tumors in A/J mice. (a) Dose-dependent inhibition of proliferation of murine and human lung cancer cell lines by rapamycin. In vitro, rapamycin only modestly inhibits proliferation of IO33 cells relative to other A/J-derived lung adenocarcinoma cell lines (CL30, CL25, and CL13) and human lung cancer cells (H1155). (b) Rapamycin inhibits mTOR in IO33 cells in vitro. mTOR inhibition was evaluated by immunoblotting analysis of cells treated with rapamycin for 2 h using antibodies specific for mTOR substrates, phospho-S6 and total 4E-BP1. (c) Syngeneic IO33 cells form invasive lung tumors in A/J mice when injected via tail vein. A representative whole mount of A/J lungs and heart 2 wk after tail vein injection with 106 IO33 cells is shown. Note multi-focal lung tumors and invasion into the ventricular wall. (d) Rapamycin inhibits mTOR in vivo, as assessed by IHC analysis of phospho-S6 in normal lung (NL) and IO33 lung tumors (TU) in A/J mice. (3.21 MB TIF) [file pone.0005061.s003.tif]
